# A Descriptive Qualitative Study of Older Persons and Family Experiences with Extreme Weather Conditions in Northern Thailand

**DOI:** 10.3390/ijerph20126167

**Published:** 2023-06-18

**Authors:** Piyatida Junlapeeya, Thaworn Lorga, Somporn Santiprasitkul, Asawinee Tonkuriman

**Affiliations:** 1 Department of Community Nursing, School of Nursing, Mae Fah Luang University, Thasud, Mueang Chiang Rai, Chiang Rai 50700, Thailand; 2 Faculty of Nursing, Chiang Mai Rajabhat University, Mae Hong Son Campus, Pang Mu, Mueang Mae Hong Son, Mae Hong Son 58000, Thailand; thaworn.lorga@gmail.com; 3 Department of Adult and Elderly Nursing, School of Nursing, Mae Fah Luang University, Thasud, Mueang Chiang Rai, Chiang Rai 50700, Thailand; somporn@mfu.ac.th; 4 Department of Mental Health and Psychiatric Nursing, School of Nursing, Mae Fah Luang University, Thasud, Mueang Chiang Rai, Chiang Rai 50700, Thailand; asawinee.ton@mfu.ac.th

**Keywords:** extreme weather, adaptive ageing, health, focus group

## Abstract

Extreme weather can cause ill health in older persons due to a direct thermal effect on the body’s thermoregulation and difficulties in maintaining a healthy lifestyle and accessing the health services they need. To understand experiences in relation to their exposures to extreme weather and how they responded to such weather conditions, including cold snaps, heat and air pollution in northern Thai communities, a descriptive qualitative study was conducted to uncover details and the essence of perspectives and experiences of older persons and family members. Three focus group discussions with 15 older persons and 15 family members occurred in three communities in Chiang Rai, a northern province of Thailand. Thematic analysis was performed. Experiences of older persons and families in relation to extreme weather conditions were described in five themes: local actions against weather changes, the double challenges, awareness and reactions to weather changes, protective and comfortable living environments, and mitigation of the impacts of weather conditions. Seasonal adaptability was key for older persons to stay safe and healthy during extreme weather changes. Heat, cold snaps, and air pollution made health and daily living routine maintenance among older persons challenging, especially among those with declining health. Older persons and families employed predictive and adaptive strategies to avoid and minimise extreme weather impacts and maximise their comfort and optimal living.

## 1. Introduction

As a result of climate change due to humans—induced acceleration in recent decades, the world has experienced extreme weather conditions such as extreme temperature (e.g., heatwaves and cold snaps), extreme events (e.g., floods and storms) and fluctuations in weather conditions [1]. During these extreme weather conditions, people can be prone to ill health due to a direct thermal effect on the body’s thermoregulation and difficulty maintaining healthy lifestyles and accessing the health services they need. Studies in different regions indicate that extreme hot and cold weather conditions are associated with higher rates of dermatological, respiratory, and cardiovascular illnesses and increased mortality risk [2,3,4]. Climate change can adversely affect air quality and harm human health [5,6,7]. Older persons are more prone to the health impacts of extreme weather conditions due to declining thermoregulation efficiency and the deteriorating effects of the weather on pre-existing respiratory and cardiovascular illnesses [8,9,10]. Older persons are more likely to experience heat and cold-related illnesses. In addition, extreme weather conditions can hamper the daily living of older persons, especially those with existing functional limitations and affect disease progression and healthcare costs [11,12,13].

The upper part of northern Thailand, including Chiang Rai, has been affected by extreme weather changes. In Chiang Rai, the yearly average temperature is 31.1 degrees Celsius. The average coldest and hottest temperature over 30 years was between 19.3 degrees Celsius and 31.1 degrees Celsius in 2019. Furthermore, the provincial temperature hit a record high of 42 degrees Celsius. The coldest record was 1 degree Celsius in 1999. [14]. Daily and weekly temperature fluctuations are common in the area. Chiang Rai has also suffered from air pollution related to forest fires and the burning of agricultural wastes in cold and hot seasons. In 2019, the province had the country’s highest annual mean of Particulate Matter 2.5 (PM 2.5) (i.e., 32.0 mcg/m^3^). This figure is three times higher than the World Health Organization’s recommendation on the safe level of PM 2.5 of 10 mcg/m^3^ [15,16]. Quantitative studies conducted in 2019 investigated the effect of air quality on the death rates of people in northern Thailand. The studies found higher death rates among people experiencing infectious diseases and respiratory illnesses in changing weather [17,18]. In colder weather, there was a higher death rate among the older population [19,20]. The scarcity of research examining the impacts of heat, cold snaps, and air pollution amidst recent weather changes in northern Thailand warrants such research. Understanding the impacts of weather changes and how older persons and their families respond to such changes can lead to the development of community services to address the needs of older persons. In Chiang Rai, the population of older persons aged 60 years or older is 278,454, accounting for 21.43% of the total population [21]. Anecdotal evidence showed that community-dwelling older persons were affected by extreme weather conditions. However, the extent of these impacts was not well-documented. This study aimed to describe the experiences of older persons and their caregivers in relation to exposure to extreme weather and how they responded to such weather conditions. A better understanding of their experiences would aid us in identifying the strengths and weaknesses of their current responses and identify opportunities to improve their responses and protect their health during extreme weather conditions.

## 2. Materials & Methods

### 2.1. Design

This study was part of a large mixed-method research project. The project was sequential, starting with a qualitative exploration followed by a quantitative survey. This qualitative study was a descriptive qualitative design. Descriptive qualitative research aims to identify aspects of the participants’ experiences related to the phenomenon under study and provide detailed descriptions of each aspect. It does not adhere to certain philosophical or theoretical perspectives [22]. This design is particularly useful when researchers want to use detailed descriptions of each aspect to further develop interventions or questionnaires for subsequent quantitative studies. In this study, the researchers described aspects of older people and family experiences in detail and planned to use these detailed descriptions for further questionnaire development.

### 2.2. Participants

The participants in this study included 15 older persons and 15 family caregivers. Through heads of village health volunteers (VHVs), older persons who had lived in the areas affected by extreme weather conditions such as extreme heat, cold snaps, extreme air pollution and sudden weather changes were invited to participate in this study. A maximum variation technique was used to recruit older participants with different levels of social participation and pre-existing illnesses. Older persons who were bedbound were not included in this study as they might have had difficulty communicating with the researchers. Family caregivers of participating older persons were also invited to share their experiences. Focus group discussions were used as the researchers wanted the group members to exchange their diverse experiences, generating information with breadth and depth. In total, three focus group discussions in three different communities were conducted. Each focus group, which took place in a communal house, comprised five dyads of older persons and caregivers (totaled 10 participants per group). Each group was homogenous when considering the socioeconomic backgrounds of the older persons and their caregivers. Having older persons and their caregivers in the same group benefitted the data collection as it promoted the exchange of information between the older persons and their caregivers. The exchange between the older persons and their family members allowed them to see the differences and similarities in their viewpoints and concerns. In addition, the exchange helped fill in the otherwise missed information and clarified each other’s experience.

The focus group discussions were guided by probing questions: How did extreme weather, such as heat, cold spells, air pollution and sudden weather changes, affect you? How did you respond to them? What did your family and your community do to help you during extreme weather conditions? These probing questions were structured around the exposure and responses to extreme weather conditions. Following the participant’s responses to the probing questions, in-depth questions were then used to facilitate subsequent discussions pertaining to what, when, why, and how of the specific experiences and viewpoints. Data collection, which took place in the community, helped sensitise the researchers about physical surroundings, living environments and cultural contexts. The direct exposures of the researchers to these environments helped guide additional questions during focus group discussion and data analysis.

### 2.3. Data Analysis

Thematic analysis, guided by a constant comparison technique [23], was used for data analysis. The researchers read through the focus group discussion transcripts to familiarise themselves with the contents and how the discussions unfolded. The researchers then identified the smallest yet meaningful units of data and assigned these units open coding with a name. The potentially connected units were then grouped into sub-themes with assigned names. These sub-themes, in turn, were later connected into a theme. Thematic analysis was suitable for this study because the researchers wanted to identify aspects (i.e., themes) of the participants’ experiences and describe each aspect of their experiences in detail (i.e., sub-themes and data units). After the third focus group discussion, the researchers did not identify new themes or sub-themes. The study has therefore satisfied thematic saturation.

## 3. Results

The communities were situated along busy highways and surrounded by rice fields. The participants lived in either brick or wooden houses, mostly without air conditioning. Nine out of 15 houses were two-story or elevated houses, where the participants sought shelter when the weather became hot. The remaining houses were one-story. Most of the houses were erected on dry land with few or very few trees. The participants’ characteristics are summarised in Table 1. Women disproportionally represented the participants due to the unavailability of potential older male participants who declined participation due to other work commitments. Three participants were homebound, and nine participants had pre-existing illnesses, including asthma, chronic obstructive pulmonary disease (COPD), hypertension (HT), diabetes mellitus (DM), and dyslipidemia (DLP). Five themes emerged, including local actions against weather changes, the double challenges, awareness of and reaction to weather changes, protective and comfortable living environments, and personal mitigation of the impacts of weather conditions, with twenty-one sub-themes (Table 2).

### 3.1. Theme 1: Local Actions against Weather Changes

All participants were aware of recent weather changes and the actions taken by the local community to address the problems in the area. The older persons and their families accepted that weather conditions would continue to worsen and felt limited control over the situation. This is particularly the case of PM 2.5, where the participants believed they could not do much to eliminate the causes or prevent the consequences of PM 2.5 in Chiang Rai.

#### 3.1.1. Sub-Theme 1.1: Worsening Weather Conditions

All the participants felt the effects of changing weather conditions on their life and health. Over the past years, they reported exposure to various unhealthy weather conditions in their community. These included heat, cold spells, smog, heavy rainfalls, and sudden weather fluctuations within a day or a week. According to the participants, these weather changes have worsened each year. Some of the recollections are as follows:

*“We have experienced many weather changes. It (the weather) can be very hot and very cold. The worst time was when we got hit by heavy smog”.*

*“Two years ago, we had a very, very bad smog. My eyes were burning. I could barely breathe and could not go out”.*

*“It was very cold the past two weeks. But luckily, it lasted only 3–4 days”.*

*“We barely saw each other because we had to stay home (during the pollution)”.*

*“Last year the smog was so heavy that we could not see the road. The smog was floating on the road”.*

#### 3.1.2. Subtheme 1.2: Reinforcing No-Burning Policy in a Local Community

Despite the no-burning policy in certain months in Chiang Rai, rice straw and bush burning are still practised to clear lands for agricultural purposes. The participants stressed the importance of helping the community to reinforce and comply with the policy.

*“The village head imposed a no-burning policy in our community. Anyone who burns grasses, leaves and garbage will receive a heavy fine. Burning is allowed during certain periods of the year”.*

*“Some people still burn grasses and leaves even though we were reinforcing the no-burning policy. But the number of people who did that was much less than in the past”.*

*“Everyone cooperated with the no-burning measure and kept an eye on those who might break the rules. If we caught anyone breaking them, we reported them to the village head”.*

#### 3.1.3. Sub-Theme 1.3: Safe and Value-Added Waste Management

To reduce PM 2.5, the community was encouraged to manage waste by using it to fertilize soil instead of burning it. In one community, there was an initiative to turn waste into fertilizer, alternative fuel, and cow feeding.

*“To prevent burning, the community offers waste collection services for a minimal charge so that the waste is properly disposed of and not burned”.*

*“A company offers us some money in exchange for haystacks. I heard they use this as an alternative energy source”.*

*“We are encouraged to use leaves and agricultural waste as fertilizers instead of burning them”.*

#### 3.1.4. Sub-Theme 1.4: Communication about Weather Conditions

Community wire audio-broadcasting was mainly used for communicating the situations and advice related to PM 2.5. Communication about other weather conditions and related advice, such as heat and cold spells, was limited.

*“During the high PM 2.5 season, the village head will make a daily announcement about the air quality and how to stay safe”.*

*“We rarely hear warnings about hot and cold weather or weather changes. Most warnings are about bad air quality that affects health”.*

*“We want to know how and what to do during extreme heat and cold. This information is lacking”.*

*“There are only 78 people in a community group LINE chat, which means many people are not included in this online group application. These people usually do not have smartphones and are left out of updated community news, including those about the weather conditions. People with access to this online community group then help pass along the information about weather changes and selfcare to the others”.*

#### 3.1.5. Sub-Theme 1.5: It’s beyond Our Community

Most participants believed that they did well with existing PM 2.5 measures and that the origins of PM 2.5 were from outside their community. They expressed limited control over the PM 2.5 situation.

*“We did everything we could to prevent PM 2.5 in our community. No one burnt leaves and grasses in our community, but the sky was still covered with heavy smog. I think the smog is from outside our community”.*

### 3.2. Theme 2: The Double Challenges

The older persons described their experiences living in extreme weather conditions as double challenges. The double challenges referred to living with aged- and disease-related frailty and exposure to the negative impacts of extreme weather conditions. The challenges could then limit their living.

#### 3.2.1. Sub-Theme 2.1: Frailer and Sicker

Most of the older persons in this study reported pre-existing illnesses such as asthma, chronic obstructive pulmonary disease, hypertension, heart disease, diabetes mellitus, and osteoarthritis. They described how advanced age and chronic illness made them physically frail. Physical frailty, in turn, posed several challenges to their day-to-day living. Some of the older participants expressed this to the group.

*“I am old and have a heart disease. This makes it difficult for me to go around the community. Bad weather adds more problems to me”.*

*“As an older person, I have become physically weaker and get sick easier”.*

#### 3.2.2. Sub-Theme 2.2: Compromised Active and Healthy Living

The older persons acknowledged that ageing and declining health had already compromised their active lifestyle. Physical frailty prevented them from participating in social and financial activities and diminished their self-care ability. As an older person living in extreme weather conditions, the challenges and living restrictions could be more intense. They expressed difficulty maintaining health staying mentally and socially and financially active. The narratives below reflect their experiences.

*“It was sweltering hot. I was sweating heavily and had to rest. I stopped going to the rice field because I used to faint in the heat”.*

*“We were forced to stay at home when the weather (air) was bad. Many things were put on hold because of the weather”.*

*“You cannot work as much when the weather is hot and polluted. This somehow can affect the amount (of money) you earn”.*

*“You can be stuck at home almost the whole day. This can be boring and stressful, especially if you are used to daily routines outside”.*

*“Hot weather affects my sleep. I don’t sleep well on hot nights. By the time I fall asleep, it will pass midnight. Because of this, I need to sleep in and get up late. I don’t feel fresh”.*

#### 3.2.3. Sub-Theme 2.3: Exacerbation of Illness

Many older persons experienced health deteriorations and exacerbation of illness during extreme weather. Respiratory and allergic diseases such as COPD could be worsened and relapsed.

*“During the cold season, I feel uncomfortable and experience chest heaviness. I cannot breathe well and cannot take a full deep breath. I have to take an inhaler”.*

*“The air pollution triggered my asthma. I felt physically unwell and could not take a full deep breath. I ended up going to see the doctor for a bronchodilator”.*

*“I cannot stand the smoke. I will immediately experience shortness of breath as soon as I smell it. I think this happens because of my bad lung conditions”.*

*“I will be short of breath during hot weather. The hotter the weather is, the more shortness of breath I feel. I try to avoid overheating”.*

*“I have hypertension. I usually have a dull pain at the back of my neck when it is hot”.*

### 3.3. Theme 3: Awareness of and Reactions to Weather Changes

The older persons and their caregivers recognised the changes in weather through bodily senses, weather forecasts and public announcements. Everyone had a unique response to the change.

#### 3.3.1. Sub-Theme 3.1: Bodily Warnings

Most of the older persons relied on their bodily senses to recognise changes in the weather. According to the participants, signs such as shivering, headache, skin itches, burning eyes, feeling feverish and shortness of breath suggested the weather changes. These signs could be felt either before or during the changes began, and they prompted the older persons and their families to respond accordingly, especially during heat or cold snaps.

*“Our body can tell in advance that the weather change is coming. It is more sensitive than the weather forecast in the TV or the phone application”.*

*“I don’t have to listen to the forecast to learn about the weather. I can just look out for the signs from my wife. Her respiratory symptoms would flare up before bad weather presents itself”.*

*“My joints will ache well before the rains start”.*

*“When the cold season is approaching, I will notice a change in my body. Before the first cold day, I had a strange feeling. I cannot tell whether it was tiredness. It is more like being giddy. And the next day, it will be cold”.*

*“I will know when the weather will change—whether hot or cold. My body will be warm and then cool, just like having a fever and shivering. And I know for sure that the weather will change”.*

#### 3.3.2. Sub-Theme 3.2: Weather Forecast and Announcement

Apart from using bodily senses, some older persons and their families accessed applications and websites to learn about daily and weekly weather. This type of access was limited only to those with smartphones with internet access. In addition, daily weather announcements and warnings through community wire-audio broadcasting were used to promote awareness of weather conditions and health-related impacts. The community broadcasting was primarily for communicating the presence of PM 2.5 before the cultivation and during the bushfire season.

*“I learn from the TV that it will be colder the next day”.*

*“I learn about the weather forecast from my smartphone. It tells us how hot and cold it will be. There is no information about the air dusts though”.*

*“The announcements through the community wire-audio broadcasting were about no-burning measures only. They did not inform us about hot and cold weather conditions”.*

*“It would be good to inform the community about the air quality through the LINE application. But someone may not know how to use it or does not have a smartphone. The village head can announce this on the community broadcasting system”.*

#### 3.3.3. Sub-Theme 3.3: Unique Reaction to Weather Changes

Not everyone experienced the same signs of weather changes. Each sign was unique to every individual older person. With their past experiences, the older persons quickly recognised the signs.

*“I am okay with the cold weather. But I am more sensitive to the heat”.*

*“In the cold season, my hands and feet become cold. It takes a long time to warm them up. My hands and feet are cold, like a dead person lying in a coffin. I try to warm them up by covering them with thick clothes. If I had access to a fireplace, I would place my hands over it”.*

*“I will sweat heavily and feel like fainting in hot weather. I need to rest; otherwise, I will blackout”.*

*“I lose my appetite during the hot season. I eat much less on hot days”.*

*“In cold weather, I don’t enjoy eating. Everything tastes so bland to me, given that I usually do not have much appetite. In cold weather, foods that used to taste good become less appetizing. Hot weather doesn’t affect my eating”.*

### 3.4. Theme 4: Protective and Comfortable Living Environments

In times of weather changes, older persons strive to adapt to the changing weather by using their living environment to protect against weather-related harms and maximise comfort. The following were natural and man-made resources used by older persons to maximise adaptability throughout different seasons.

#### 3.4.1. Sub-Theme 4.1: Natural Environments

Trees, flowing water streams, shades, and flowing air were described by the older persons as protective and alleviating when they experienced heat waves and PM 2.5. These natural environments were found in their homes and yards.

*“It was extremely hot last April. I had to stay under the tree or shade during the day”.*

*“Staying near a flowing stream and in the area where the air is moving can help eliminate the discomfort from the heat. It can be quite refreshing”.*

#### 3.4.2. Sub-Theme 4.2: Man-Made Environments

Rest areas with shade and decking were used as a shelter when there was excessive heat and PM 2.5. Rest areas in the community were often built under or around the house.

*“You need to build a shade to escape the heat on a hot day”.*

*“Staying under the (elevated) house is one of the options when the temperature is soaring, and your room gets overheated (by the sun) during the day”.*

*“Windows and doors can help you control indoor weather conditions. You close the windows to block out the cold snap or pollution. You open them when you want to ventilate the room and reduce the heat”.*

#### 3.4.3. Sub-Theme 4.3: Adaptive Living Appliances

Some appliances such as electric fans, air conditioners and water heaters were described by the older persons as necessary during the hot and cold seasons and the presence of high PM 2.5. It was noted that expensive items such as air conditioners were affordable by only one family in this study.

*“When the weather is boiling, I stay in an air-conditioned room. But then you can expect a surging electricity bill”.*

*“I use the electric fan to help cool the air. You just have the fan constantly blowing on you to stay cooler”.*

#### 3.4.4. Sub-Theme: 4.4 Acceptability of Clean Room Services

A clean room at a primary care unit was proposed as a solution to the PM 2.5 crisis. Its use in practice was, however, questioned by the participants. Geographical accessibility was mentioned as a barrier to this service.

*“I heard of a clean room for safe sheltering during high PM 2.5 at the community health centre. But I think it is over two kilometers from here and I wonder how I would go there. And the fact that there is only one room makes me think that the room will be cramped with people seeking shelter there”.*

*“I may go to the clean room if it is near. But I would rather stay at my place”.*

*“I think I will consider using a clean room if it is located closer to the community”.*

*“I don’t know. I haven’t heard about the clean room for safe stay when the community is smoked”.*

*“It is impossible during the COVID-19 pandemic. It will spread the disease”.*

### 3.5. Theme 5: Personal Mitigation of the Impacts of Weather Conditions

Living in extreme weather conditions requires older persons and their families to work out strategies to manage their daily living and health effects. The analysis of their narratives identified at least six strategies used by the participants.

#### 3.5.1. Sub-Theme 5.1: Having Good Health

Having good health was believed to counterbalance the effects of worsening weather conditions. One of the participants, for example, mentioned the need to regularly follow healthy lifestyles and stay healthy to stay strong amidst the weather extremes.

*“You have to keep yourself healthy. Eat well”.*

*“Keep your body strong. Make sure you won’t fall ill when the weather changes”.*

*“It’s easy. You put your clothes on when the weather is cold and turn on an electric fan when it is hot”.*

*“You simply take a rest. You do nothing, just sit there. You must not walk back and forth. You need to station yourself somewhere so that you won’t tire your body”.*

#### 3.5.2. Sub-Theme 5.2: Prediction

As mentioned, older persons relied on their bodily senses, weather forecasts, and announcements to learn about weather situations. Being aware of the severe upcoming weather conditions helped older persons and their families to predict and respond to the situation appropriately. One of the participants said:

*“I know the cold season is approaching because I will notice a change in my body. Before the first cold day, I had a strange feeling. I cannot tell whether it was tiredness. It is more like being giddy. And the next day, it will be cold”.*

*“I learn from the TV that it will be colder the next day”.*

#### 3.5.3. Sub-Theme 5.3: Prevention

Older persons use simple measures to prevent the conditions and illnesses caused by extreme weather conditions. These included, but were not limited to, wearing adequate clothing and blankets, sitting by the fire to stay warm, wearing a face mask and shutting the window to block off the PM 2.5, having a fan on, taking a bath, and limiting the activities to reduce body heat. The older persons said:

*“It’s easy. You put your clothes on when the weather is cold and turn on an electric fan when it is hot”.*

*“If the weather is too hot, cold, or smoggy, I won’t go out or work outside”.*

*“I am wearing a face mask on a smoggy day, especially when riding a motorcycle; otherwise, your nose will be dry”.*

*“I close the doors and windows when the outside air is bad. Turn on the fan to circulate the indoor air. This can help, even for a little. It is better than doing nothing”.*

#### 3.5.4. Sub-Theme 5.4: Seasonal Adaptability

Weather changes interfered with their daily routines. The older persons needed to adjust how they go about their routines, such as gardening, and exercise completed. Certain planned activities were sometimes halted by weather conditions. In our study, this strategy was applied to an exercise program held regularly. This contingency plan for exercise was often used during the months of high PM 2.5 concentration, where the older persons were instructed to come to the program or stay home based on the level of PM 2.5.

*“I move my gardening to the early morning, so I won’t feel much heat in the evening”.*

*“I have to adjust my routines to fit with the weather conditions. If it rains, I will put off outdoor activities and spend time doing household chores”.*

*“During a cold season, I reschedule a shower from late evening to 3 PM to avoid getting too cold and getting ill”.*

*“We may plan for a group outdoor exercise every day in the evening. But we need to have plan B by doing it indoors instead if the area is hit by PM 2.5”.*

*“I carry an insulated hot herbal drink bottle with me wherever I go for instant drinks. This is to keep myself hydrated and warm”.*

*“I soak a towel in cooling balm water, place it in a fridge until it gets very cold and rub the towel over my body to reduce the heat”.*

*“Spraying water into the air, onto the ground and around us can help restore freshness in extreme heat”.*

#### 3.5.5. Sub-Theme 5.5: Emergency Management

The emergency mentioned by the older persons concerned individual and public health emergencies. The older persons described how they managed their health problems caused by weather changes and how the community responded to air pollution when it was declared dangerous and hazardous.

*“I went to the clinic for a nebulizer because my breathing was troubled (by the PM 2.5)”.*

*“If the air is bad, I may need to go to the safe room at the community health centre”.*

*“Her skin swelled badly as a reaction to the heat, and she needed to go to the hospital for an injection to relieve the symptom”.*

#### 3.5.6. Sub-Theme 5.6: Keeping Busy

After all, living a restricted life in a hazardous environment could take a toll on the mental well-being of older persons. Keeping oneself busy with household activities, work, and hobbies helped relieve the stress of being restricted from daily routines.

*“I tell myself that thing (weather conditions) will just be like this. I cannot be too worried about it anymore. Live with it. Keep doing what you do if you can”.*

*“You couldn’t do much during bad weather. You had to keep yourself busy. Otherwise, you would get stressed”.*

## 4. Discussion

The older persons’ experiences regarding the impacts of extreme weather conditions were represented in five themes: local actions against weather changes, the double challenges, awareness and reactions to weather changes, protective and comfortable living environments, and personal mitigation of the impacts of weather conditions. The findings reflected the awareness of older persons and their families about local weather changes, their effects on the health and living of the older persons, and actions to promote adaptive and optimal living in the face of these challenging conditions. According to the participants, Chiang Rai is vulnerable to extreme weather conditions. In line with the recent study’s findings, older persons, especially those who are physically frail and financially disadvantaged, are susceptible to physical vulnerability due to extreme weather conditions [3]. Older persons reported frequent ill physical health and diminished physical and social functions during weather changes, whereas some reported diminished economic productivity [24]. Physical senses were used as indicators for weather changes. Despite the availability of information technology on weather and the impacts of weather changes on health, older persons rarely access this technology. Access to information technology among older persons has been reported as low, especially among older persons with low literacy and limited access to technologies such as smartphones. While a study stated that specifically designed applications would impact older persons on accessibility to the local environment, potentially leading to increased walking and social engagement [25]. In weather changes, older persons strive to adapt to the changing environments using their living environments to protect against weather-related harms and maximise comfort [2,26]. Protective and comfortable living environments for counteracting the weather are the issue of interest among researchers, policymakers, and service providers internationally. Success in reducing the impact of climate and environmental variations on the health of the elderly depends on many factors, including community and management factors [2,3,10,16,24,26,27]. In Thailand, however, these developments are still embryonic. Responses to mitigate weather impacts appeared to rely on personal and family capabilities. These responses are often inadequate in addressing older persons’ health and living needs, which can, in turn, compromise their health. To protect the health of older persons during extreme weather conditions, several national and local policies, such as the Clean Room policy, have been advocated [28]. But in practice, some of these policies have been considered impractical and unfeasible due to costs, lack of contextual appropriateness and acceptability of the older persons, as consistent with a study that pointed out the necessity to address elder-specific needs in developing adaptation strategies to climate change [8]

Some more affordable services, such as weather communication, have not yet been effectively utilised. Its effects are, therefore, not fully realised. Weather and health communications are one of many promising means to promote weather-related resilience. This is in accordance with the recommendation of the World Health Organisation (WHO), which highlights the need for additional communication efforts to ensure information is accessible and at the appropriate level for older persons [16].

The overarching theme, “Adaptive and optimal living”, captured the overall experiences of older persons and their families when facing and coping with extreme weather conditions. Adaptive and optimal living suggested the need for older persons to adapt their environments and routines so that their housing was still livable, and they remained safe and healthy amidst health-exacerbating extreme weather conditions. In this study, optimal living concerns the interplay between personal livelihood, environmental safety, and individual health. Optimal living requires older persons and their families to balance the benefits and harms of engaging in certain activities in the presence of extreme weather. Optimal living, in turn, necessitated modifications to physical living conditions and adjustments to daily routines

The findings from this study have several implications for improving the health and well-being of older persons living in extreme weather-prone areas. These included promoting awareness about weather changes, fostering personal adaptive capability, and strengthening and re-designing community services. Awareness raising should cover local weather changes such as heat, cold snaps, and air pollution as well as their impacts on the health and living of older persons. In fostering personal adaptive capability, older persons recognise one’s strengths and weaknesses, create protective and comfortable living environments and employ seasonal and daily adaptability strategies. Innovative information, health and social services should be implemented to strengthen existing community services. The findings from our study cannot be readily generalised to other settings due to the use of small sample size and a purposive sampling technique in only three local communities. This represents a limitation of our study. Careful considerations should be given when applying our findings to other settings.

## 5. Conclusions

Heat, cold snaps, and air pollution adversely affected the health and living of older persons living in Chiang Rai, Northern Thailand. Adaptive and optimal living strategies were used by older persons and their families when facing and coping with such extreme weather conditions. Optimal living was the interplay between personal livelihood, environmental safety, and individual health, where older persons needed to make careful decisions based on the risks and benefits of engaging in certain activities in the face of apparent harm from extreme weather. To stay safe and healthy, older persons needed to adjust their housing conditions and routines. Awareness of weather changes, its impacts on health and living, and one’s strengths and weaknesses fostered personal adaptive capabilities in older persons during extreme weather conditions. Protecting the health and well-being of older persons during extreme weather should consider promoting awareness about weather changes and fostering personal adaptive capability.

## Figures and Tables

**Table 1:** Demographics of the older persons (N = 15) and their family members (N = 15).

	Gender		
Characteristics	Female	Male	Total
	*n*	*n*	
**Older persons**			
**Age (Years)**			
60–70	11	-	11
>70	2	2	4
Total	13	2	15
**Levels of social participation**			
homebound	1	2	3
socially active	12	-	12
Total	13	2	15
Pre-existing illnesses *			
Asthma	1	1	2
COPD	1	-	1
HT	2	1	3
DM	2	1	3
DLP	1	1	2
Heart disease	-	1	1
Osteoarthritis	1	-	1
Total	8	5	13
**Family members**			
**Age (Years)**			
<60	3	-	3
60–70	7	2	9
>70	2	1	3
Total	12	3	15

* one participant had more than one disease.

**Table 2:** The overarching theme, themes, sub-themes, and examples of open coding.

Overarching Theme	Themes	Sub-Themes	Examples of Open Coding
Adaptive and optimal living	1. Local actions against weather changes	1.1 Worsening weather conditions	*“We have experienced many changes in weather in recent years. It [the weather] can be very hot and very cold. The worst time was when we got hit by heavy smog”.*
		1.2 Reinforcing no-burning Policy in a local community	*“The village head imposed a no-burning policy in our community. Anyone who burns grasses, leaves and garbage will receive a heavy fine. Burning is allowed during certain periods of the year”.*
		1.3 Safe and value-added waste management	*“Some measures encourage people to make money from wastes. Instead of burning them. People sell haystacks to a merchant who processes these wastes into fertilisers”.*
		1.4 Communication about weather conditions	*“We rarely hear warnings about hot and cold weather or weather changes. Most warnings are about bad air quality that affects health”.*
		1.5 It’s beyond our community	*“We did everything to prevent PM 2.5 in our community. No one burnt leaves and grasses in our community, but the sky was still covered with heavy smog. I think the smog comes from outside our community”.*
	2. The double challenges	2.1 Frailer and Sicker	*“As an older person, I have become physically weaker and get sick more easily”.*
		2.2 Compromised Active and healthy living	*“It was sweltering hot. I was sweating heavily and had to rest. I stopped going to the rice field because I used to faint in the heat”.*
		2.3 Exacerbation of illness	*“The air pollution triggered my asthma. I felt physically unwell and could not take a full deep breath. I ended up going to see the doctor for a bronchodilator”.*
	3. Awareness of and reactions to weather changes	3.1 Bodily warnings	*“Our body can tell in advance that the weather change is coming. It is more sensitive than the weather forecast in the TV or the phone application”.*
		3.2 Weather Forecast and Announcement	*“I learn from the TV that it will be colder the next day”.*
		3.3 Unique reaction to weather changes	*“In the cold season, my hands and feet become cold. It takes a long time to warm them up. My hands and feet are cold, like a dead person lying in a coffin. I try to warm them up by covering them with thick clothes. If I had access to a fireplace, I would place my hands over it”.*
	4. Protective and comfortable living environments	4.1 Natural environments	*“It was extremely hot last April. I had to stay under the tree or shade during the day”.*
		4.2 Man-made environments	*“You need to build shade to escape the heat on a hot day”.*
		4.3 Adaptive living appliances	*“When the weather is boiling, I am forced to stay in an air-conditioned room. But then you can expect a surging electricity bill”.*
		4.4 Acceptability of clean room services	*“I heard of a clean room for safe sheltering during high PM 2.5 at the community health centre. But I think it is over 2 km from here and I wonder how I would go there. And the fact that there is only one room makes me think that the room will be cramped with people seeking shelter there”.*
	5. Personal mitigation of the impacts of weather conditions	5.1 Having good health	*“Keep your body strong. Make sure you won’t fall ill when the weather changes”.*
		5.2 Prediction	*“I know the cold season is approaching because I will notice a change in my body. Before the first cold day, I had a strange feeling. I cannot tell whether it was tiredness. It is more like being giddy. And the next day, it will be cold”.* *“I learn from the TV that it will be colder the next day”.*
		5.3 Prevention	*“It’s easy. You put your clothes on when the weather is cold and turn on an electric fan when it is hot”.*
		5.4 Seasonal adaptability	*“We may plan for a group exercise every day in the evening. But we need to have plan B by doing it indoors if the area is hit by PM 2.5”.*
		5.5 Emergency management	*“I went to the clinic for a nebulizer because my breathing was troubled (by the PM 2.5)”.*
		5.6 Keeping busy	*“You couldn’t do much during bad weather. You had to keep yourself busy. Otherwise, you would get stressed”.*

## Data Availability

The data will be available upon request.
